# Licury Cake in Diets for Lactating Goats: Qualitative Aspects of Milk and Cheese

**DOI:** 10.3390/ani13010035

**Published:** 2022-12-22

**Authors:** Fernanda G. Ferreira, Laudí C. Leite, Henry D. R. Alba, Douglas dos S. Pina, Stefanie A. Santos, Manuela S. L. Tosto, José E. de Freitas Júnior, Carlindo S. Rodrigues, Bruna M. A. de C. Mesquita, Gleidson G. P. de Carvalho

**Affiliations:** 1 Department of Animal Science, Universidade Federal da Bahia, Av. Milton Santos, 500, Ondina, Salvador 40170110, Brazil; 2 Department of Animal Science, Universidade Federal do Recôncavo da Bahia, Cruz das Almas 44380000, Brazil; 3 Institute of Agricultural Sciences, Universidade Federal de Minas Gerais, Montes Claros 39404547, Brazil

**Keywords:** by-product, fatty acid profile, goat, licury cake, Minas Frescal cheese, small ruminant

## Abstract

**Simple Summary:**

Feed costs are high in feedlot systems. To reduce these costs, cheaper alternatives are studied. The use of licury cake (LC) is an economical alternative because it can compete (economically and nutritionally) with common ingredients used in animal feed. However, the ruminant has different feed degradability and digestibility mechanisms, promoted by symbiosis with microorganisms in the rumen that can affect the physicochemical characteristics of the final product. We studied dietary LC inclusion, at levels of 0, 67, 133 and 200 g/kg of dry matter (DM), to evaluate its impact on qualitative aspects of milk and cheese. The inclusion of LC up to 133 g/kg DM is recommended for the diet of lactating goats whose milk will be used in the production of Minas Frescal cheese.

**Abstract:**

The study was carried out to evaluate the effects of licury cake (LC) inclusion in diets for lactating goats on milk chemical composition and fatty acid profile, and physicochemical composition and sensory attributes of Minas Frescal cheese. Twelve lactating goats were used (eight Saanen and four Anglo Nubian goats, with 35 ± 5 days in lactation and average body weight of 37.93 ± 9.22 kg), distributed in a triplicate 4 × 4 Latin Square design. The increasing levels of LC studied were: 0, 67, 133 and 200 g/kg of dry matter (DM). The analyses were adjusted using orthogonal polynomial contrasts, in which the probability level of 5% was considered. For sensory attributes, principal component analysis was performed. The LC inclusion promoted the reduction in moisture (*p* = 0.013) and mineral matter (*p* < 0.001) content in cheese. The ether extract content in cheese showed a quadratic effect (*p* = 0.021). Lauric acid showed a linear (*p* = 0.002) increasing effect, and myristic (*p* < 0.001) and rumenic (*p* = 0.018) acids showed quadratic effects. The sensory attributes analysis in cheese indicated that the inclusion of 133 and 200 g/kg of LC improve its texture. Flavor was improved with the inclusion of 67 and 133 g/kg. The inclusion of up to 133 g/kg of LC in the diet of lactating goats is recommended because it promotes improvements in flavor and texture of Minas Frescal cheese.

## 1. Introduction

The largest goat herd in Brazil is found in tropical regions where goat breeding is predominantly in extensive production systems. In these systems, the feed base is native forage, which is affected in terms of availability and quality (high lignin and low protein levels) depending on the time of year, negatively affecting the productive performance of the animals [1].

Breeding in intensive or semi-intensive systems is a strategy used to avoid low feed availability. The quality of the feed is superior in these systems, avoiding variations in milk production throughout the year. However, in these systems it is necessary to use large amounts of concentrate, increasing production costs [2]. As an alternative to reduce feed costs (≥60%) promoted by the use of traditional feeds (soybean meal and corn), the use of alternative feeds is recommended, since these have less commercial value, therefore improving the cost/benefit ratio [3].

Among the alternative feeds with potential to be used in ruminants feeding, the by-products of the processing of oilseed grains and palm fruits have been found in recent years. Licury (*Syagrus coronata*) is a palm well adapted to arid and semiarid regions, such as the Caatinga in Brazil. Its use is aimed at human food, construction, cosmetics, hygiene, biofuels and medicinal products [4,5]. Licury cake is obtained after extracting the oil from the kernel. It has competitive levels of crude protein (CP; 222 g/kg dry matter (DM) basis), ether extract (EE; 61 g/kg DM) and neutral detergent fiber (NDF; 486 g/kg DM) for ruminant nutrition [6].

The intake, digestibility, growth performance, and metabolism in sheep [7], goats [8] and cattle [3,9] supplemented with LC was evaluated. From these studies, the ideal levels of inclusion of 420 g/kg DM of this by-product were seen to promote positive results for meat production and 400 g/kg DM for milk production in dairy cows.

Licury cake is generally included in the diet as a substitute for corn and soybeans, mainly soybean meal, which can be up to 100% substituted with the LC inclusion. Dry matter intake decreases with LC inclusion greater than 7–9% in cows, steers, goat kids, and lambs in feedlots. Furthermore, the inclusion linearly decreases digestibility. These changes affect ingestive behavior, feed efficiency, and nitrogen retention; consequently, the productive performance of these meat ruminants is affected after a certain level of LC inclusion in the diet [6,9,10,11].

Physicochemical attributes and sensory characteristics in beef cattle are not affected by LC inclusion in the diet [12,13]. However, in small ruminants, in feedlot lambs and goat kids, changes are observed in some meat characteristics, such as increased lightness and redness, decreased shear force, and decreased fat and protein contents. Furthermore, with increasing LC inclusion levels, alterations of the fatty acid profile are observed, mainly with quadratic effects on saturated, monounsaturated, and polyunsaturated fatty acids, as well as on atherogenicity, thrombogenicity and hypocholesterolemic and hypercholesterolemic indexes [6,14].

In grazing dairy cows, the observed effects of the LC inclusion in the diet are not usually significant, probably due to the low proportion that the supplement represents in the diet and the amount of LC in this supplement [3]. In this sense, it is imperative to study the inclusion of different levels of LC in the diet of confinement dairy ruminants (mainly small ruminants due to the pronounced effects of this inclusion on beef animals), since feeding in confinement systems represents a higher proportion of production costs.

Furthermore, to increase the profitability of the farm, the products obtained (milk, meat, etc.) are transformed into others with higher added value (cheese, yogurt, etc.) and it is important to evaluate the effect of LT on the physicochemical and sensory characteristics of these products.

Therefore, it is hypothesized that there is an ideal level of inclusion of LT in the diet of lactating goats that can promote improvements in milk chemical composition and fatty acid profile, and physicochemical composition and sensory attributes of Minas Frescal cheese from feedlot lactating goats. In this sense, the objective of the current study was to evaluate the effects of the licury cake inclusion in diets for lactating goats on milk chemical composition and fatty acid profile, and physicochemical composition and sensory attributes of Minas Frescal cheese.

## 2. Materials and Methods

### 2.1. Ethics Committee and Experiment Location

The experiment followed animal welfare rules, and thus the project was approved (approval no. 73/2018) by the Ethics Committee of the Use of Animals (CEUA) at the Federal University of Bahia (UFBA). The experiment was conducted in the goat breeding section of the “Entre Rios” Experimental Farm—UFBA, located in the municipality of Entre Rios, Brazil (11°56′31″ S, 38°05′04″ W, 162 m above sea level).

### 2.2. Animals, Experimental Design and Management

**Figure 1:**
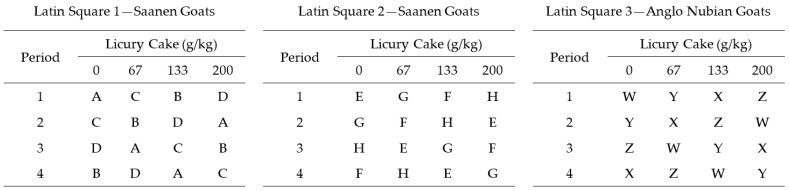
Experimental design considering three parallel Latin Squares of four periods and four treatments (animals: A, B, C, D, E, F, G, H, W, X, Y, and Z).

Twelve multiparous lactating goats (eight Saanen and four Anglo Nubian goats; average weight of 46.9 ± 9.4, averaging 105 ± 5 days in milk; and with an average production of 1.5 ± 0.4 kg/day) were used. Animals came from the goat area of the “Entre Ríos” Experimental Farm—UFBA, and were selected considering the uniformity in breed anatomical characteristics, body weight, days in milk, and milk production. Goats were distributed in a triplicate 4 × 4 Latin square design (Figure 1).

Before the beginning of the experiment, fifteen days were used to adapt the animals to the facilities, milking management and concentrate content of diets. The study was divided into four periods of 14 days each. Therefore, the total duration of the study was 56 days. Each period was divided into ten days for the adequacy of the animals to the by-product in the diets and four days for the data and samples collection.

The goats were housed in 1.5 m^2^ individual pens; equipped with drinker and feeding trough. Water was provided *ad libitum*. Feed was supplied with daily adjustments to allow around 10% refusals.

Diets were formulated according to the NRC [15] to meet the requirements for maintenance and milk production of lactating goats. The experimental diets consisted of increasing inclusion rates of LC: 0, 67, 133 and 200 g/kg DM (Table 1) and fed as a total mixed ration, twice daily (08:00 h and 15:00 h). A forage:concentrate ratio of 40:60 was adopted, with maize silage used as the forage.

Hand milking (once a day) was performed at 07:00 h, after pre-dipping with a 0.5% glycerin iodine solution. After milking, post-dipping was performed using a 0.5% glycerin iodine solution. Hygiene procedures were followed to avoid mammary gland infections. The average body weight of the goats was obtained after the pre-adaptation period and on the first and last day of each period (day 1 and 14). The animals were weighed before supplying the diets, in the morning and with the help of an electronic scale.

Feed intake was calculated by the difference between the amount of the component present in the diet supplied and in refusals.

### 2.3. Chemical Analysis

Samples of experimental diets (ingredients, concentrate, and forage) and refusals were collected the last seven days of each period. Refusals (all food that was not eaten by animals) were collected before the first feeding; the sampling of the ingredients was carried out before the elaboration of the diets, ensuring the collection of a representative sample, and the concentrate and forage were collected once a day in the morning. Each sample was pre-dried in a forced ventilation oven (55 °C for 72 h). Afterwards, samples were ground in a Wiley-type blade mill to 1 mm for the determination of the chemical composition and to 2 mm for the determination of non-digestible neutral detergent fiber. Dry matter (934.01), ashes (930.05), crude protein (CP, 981.10), and ether extract (EE, 920.39) were determined following the methodology proposed by the Official Association of Chemical Analysis [16].

Neutral detergent fiber (NDF) and acid detergent fiber (ADF) determinations were performed according to Van Soest et al. [17]. FDN and FDA were corrected for ash and protein (NDFap and ADFap) following the methodology described by Mertens [18] and Licitra et al. [19], respectively. Lignin was determined according to the AOAC 973.18 method [20].

The estimates of non-fibrous carbohydrates (NFC) were made according to the formula proposed by Hall [21] and Detmann and Valadares Filho [22], considering, or not, the use of urea in the diet formulation.

The non-digestible neutral detergent fiber (iNDF) was determined by incubating the samples (In Situ method, using 100 g m² TNT bags) according to the methodology described by Reis et al. [23]. The potentially digestible neutral detergent fiber (pdNDF) was obtained by the difference between NDFap and iNDF.

### 2.4. Chemical Composition of Milk

Milk production per animal and per day was recorded and sampled during the last four days of each period. One representative sample, approximately 40 mL per animal (Totaling 48 samples per period), was stored in a plastic bottle containing the preservative 2-bromo-2-nitropropane-1,3-diol (bromopol). These samples were analyzed in the Bentley Instruments^®^ Infrared Analyzer (Bentley Instruments Inc., Curitiba, Paraná, Brazil) to determine the contents of protein, fat, lactose, urea nitrogen and total solids. Somatic cell counting was performed using the Bentley Instruments^®^ Soma-count-500 device (Bentley Instruments Inc., Curitiba, Paraná, Brazil). These analyses were performed in the laboratory of the Clinica do Leite ESALQ/USP, Piracicaba—SP. To obtain the content of milk components (g/day), the percentage of each component (fat, protein, lactose, and total solids) was multiplied by the volume of milk produced (g/day). The milk and cheese were weighed using a computerized balance Toledo Prix 4 Uno with a printer.

### 2.5. Fatty Acid Profile of Milk

Milk samples intended for fatty acid analysis were stored in airtight containers and frozen at −20 °C. The fat of these samples was extracted with the organic solvent hexane. Fatty acid methylation was carried out in two steps, using basic (sodium methoxide) and acid (acetyl chloride) catalysts [24].

The quantification of fatty acid methyl esters was performed by gas chromatography (Focus GC-Thermo Scientific; Thermo Fisher Scientific; São Paulo, Brazil), with a flame ionization detector (CG-DIG) and SP-2560 capillary column (Supelco, 100 m × 0.25 mm × 0.2 μm). The gas flow was 1.5 mL/min of H_2_ (carrier gas). The detector and injector temperatures were set at 250 °C. The temperature gradient used was had an initial temperature of the column established at 70 °C, maintained for 4 min, and then the temperature was raised to 175 °C at a rate of 13 °C per minute, maintained at this temperature for 27 min, increasing again at 215 °C, at a rate of 4 °C per minute, maintaining this temperature for 31 min [24]. The identification of fatty acid methyl esters was based on retention times of FA 275 standards (GLC-674, Nu-Chek Prep, Inc.).

Total saturated (SFA), monounsaturated (MUFA), and polyunsaturated (PUFA) fatty acids were calculated, as well as the ratios between omega-6 (ω-6) and omega-3 (ω-3) fatty acids.

The nutritional quality of the lipid fraction was evaluated by indices, based on fatty acid composition data, using the formulas proposed by Ulbrich and Southage [25], through the following calculations:

Atherogenicity index (AI) = [12:0 + (4 × 14:0) + 16:0]/(Σ ω-6 + Σ ω-3 + ΣMUFA ω-9)

where: 12:0 = lauric acid, 14:0 = myristic acid, 16:0 = palmitic acid, Σ ω-6 = sum of omega-6 polyunsaturated fatty acids, Σ ω-3 = sum of omega 3 polyunsaturated fatty acids, and ΣMUFA ω-9 = sum of omega 9 monounsaturated fatty acids.

Thrombogenicity Index (TI) = (14:0 + 16:0 + 18:0)/(0.5 × ΣMUFA) + (0.5 × Σ ω-6) + (3 × Σ ω-3) + (Σ ω-3/Σ ω-6)

where: 18:0 = stearic acid and ΣMUFA = monounsaturated fatty acids.

The hypocholesterolemic:hypercholesterolemic fatty acid ratio (h:H) was estimated according to Bessa [26] and Santos-Silva et al. [27]:

h:H = (C18:1 cis9 + C18:2 n-6 + 20:4 n-6 + C22:5 n-3)/(C14:0 + C16:0).

where: C18:1 cis9 = oleic acid, C18:2 n-6 = linoleic acid, 20:4 n-6 = arachidonic acid, and C22:5 n-3 = docosapentaenoic acid.

### 2.6. Cheese Production and Yield

Milk destined for cheese production was collected in the last four days of each period. This was weighed, sieved, and stored in hermetic containers at −20 °C until the cheese was made.

For the preparation of the cheese, the milk was slowly thawed in the refrigerator. The elaboration of the Minas Frescal cheese was carried out following the sanitary norms recommended in Ordinance N° 326 of the Ministério da Saúde, which guarantees safety and quality in food manufacturing [28].

Initially, the milk was pasteurized at 60 °C for 30 min. For this process, an electric gas stove, 4 pots and a thermometer were necessary to pasteurize the milk collected by animal/treatment/period. Immediately, it was cooled in an ice bath, 38 °C. Minas Frescal cheese was produced according to the method described in Malheiros et al. [29], using as ingredients potassium chloride (0.02%; Rica Nata, Minas Gerais, Brazil), cultures provided by skimmed natural yogurt (1.8%; Nestlé), sodium chloride (0.8%; Sal Lebre), and liquid coagulant (CHY-MAX^®^). The curds were placed in cylindrical forms (perforated and sterilized) and kept at room temperature, turning every 1 h until the final dripping. Four cheeses were obtained per treatment.

Cheeses were packed and kept refrigerated at 4 ± 1 °C for approximately 24 h, until the physicochemical and sensory analyses. Afterwards, cheese samples were collected for physicochemical analysis, including humidity (gravimetric method).

For the chemical composition, cheese samples were lyophilized in LV2000^®^ lyophilizer (Equipment’s Terroni Ciencianos, São Carlos, SP, Brazil). Ash (930.05), CP (981.10) and EE (920.39) were determined according to AOAC methods [11]. Cheese yield was calculated according to El-Gawad and Ahmed [30]:

Yield = [(0.93 G + C − 0.1) × 1.09 × 100]/(100 − U)

where: G is milk fat (%), C is casein (%), and U is moisture (%).

The milk and cheese were weighed using a computerized balance Toledo Prix 4 Uno, Toledo do Brazil, São Paulo, Brazil with a printer.

### 2.7. Sensorial Analysis

Sensory analysis was performed by applying a questionnaire [31], in which five sensory attributes were evaluated: color, odor, flavor, texture, and global acceptance. The sensory analysis was evaluated according to the following nine-point scale: dislike extremely (1), dislike very much (2), dislike moderately (3), dislike slightly (4), neither like nor dislike (5), like slightly (6), like moderately (7), like very much (8), and like extremely (9). The test was carried out with a panel of 103 untrained tasters, previously selected as consumers of dairy products, who did not suffer from allergies and who were interested in participating in the sensory analysis. Of the 103 tasters, 67.3% were women and 36.7% men, 85.1% were between 18–30 years old, 8.6% between 31–40 years old, 4.9% between 41–50 years old, and 1.0% between 51–60 years old. The frequency of consumption of goat’s cheese by the tasters was: 90.6% consume it rarely, 8.9% sporadically, and 1% frequently.

Each taster received four samples of fresh cheese (1.5 × 1.5 × 1.5) corresponding to the four inclusion levels of LC (0, 67, 133 and 200 g/kg DM), salt and water biscuit, and mineral water. It was recommended that between samples. The biscuit and water have the function of cleaning the taste buds; therefore, the previous sample would not interfere with the sensory analysis of the later sample [32]. Samples were coded with three random digits and supplied in sealed pots to maintain sensory characteristics. The evaluation was carried out in the morning, between 09:00 and 12:00 h.

### 2.8. Statistical Analysis

For the analyses, SAS (Statistical Analysis System) statistical software version 9.2 [33] was used. The variables of intake, digestibility, feeding behavior, milk production, and nitrogen metabolism were assessed according to a triplicated 4 × 4 Latin Square. The mathematical model below was applied:

Ŷ_ijkl_ = μ + LS_i_ + A(LS_i_)_j_ + P_k_ + LC_l_ + LS_i_ × LC_l_ + ε_ijkl_;

where Ŷ_ijkl_ = dependent variable; μ = overall mean; LS_i_ = fixed effect of the Latin Square (i = 1, 2 and 3); A(LS_i_)_j_ = random effect of the animal into the Latin Square (j = 1, 2, 3 and 4); P_k_ = random effect of the period (k = 1, 2, 3 and 4); LC_l_ = effect of the LC inclusion level (l = 0, 67, 133 and 200 g kg^−1^); LS_i_ × LC_l_ = fixed effect of the interaction between Latin Square and LC inclusion level; and ε_ijkl_ = random experimental error associated with each observation, with NID ~ (0, σ2) assumption.

Furthermore, the effect of the LC inclusion level was evaluated using Orthogonal Polynomial Contrasts to determine the linear (−3, −1, +1, +3) and quadratic (+1, −1, −1, +1) effects. For all the evaluations, the level of 5% probability of type I error (*p* ≤ 0.05) was considered. No interaction between treatment and racial group was observed for any of the variables studied.

The sensory analysis constituted a set of multivariate data that were arranged in a matrix (412 × 6) and interpreted using principal component analysis (PCA). For this analysis, SAS software version 9.4 (SAS Institute Inc.; Cary, NC, USA) was used with data centered on the mean.

No breed effect or interaction between treatment and breed group was observed for any of the variables studied.

## 3. Results

### 3.1. Nutritional Components Intake and Milk Chemical Composition

There were no differences in the nutritional components’ intake or in milk composition (*p* > 0.05) promoted by increasing levels of LT inclusion in the diet of lactating goats (Table 2). The average DM intake was 1.61 kg/day and of protein was 0.3 kg/day. The mean values observed for fat, protein, lactose, and total solids were 39.4; 33.9; 45.5, and 126.5 g per day, respectively.

### 3.2. Fatty Acid Pofile of Milk

The inclusion of LC in diets for lactating goats promoted increasing concentrations of lauric (12:0), myristic (14:0) and myristoleic (14:1) fatty acids in goat milk (*p* < 0.05). On the other hand, heptadecanoic acid (17:0) showed a linear decreasing effect, whereas rumenic acid (18:2cis9trans11) showed quadratic behavior, with a minimum content of 0.29 mg/100 mg with the inclusion of 10.1 g of LC/kg DM in the diet (Table 3).

The licury cake inclusion had no effect on polyunsaturated (*p* > 0.05), monounsaturated (*p* > 0.05), and saturated (*p* > 0.05) fatty acid proportions (Table 4), with mean values of 4.19, 25.10, and 64.80 g/100 mg, respectively. Furthermore, there was no influence on the atherogenicity (*p* > 0.05) and thrombogenicity (*p* > 0.05) indexes (Table 4) with mean values of 2.41 and 3.18, respectively.

### 3.3. Quality of Minas Frescal Cheese

The inclusion of LC gradually reduced the ash content (*p* < 0.001) of the cheese. On the other hand, moisture and ether extract showed quadratic behavior with a minimum value of 53.89% with the inclusion of 116.7 g of LC/kg DM (*p* = 0.001) and a maximum value of 47.04% with the inclusion of 154.72 g of LC/kg DM (*p* = 0.021), respectively. Crude protein concentration and yield were not affected (*p* > 0.05) by the inclusion of LC in the diet of lactating goats (Table 5).

### 3.4. Sensorial Analysis

Principal components analysis allows us to observe the distribution of treatments on specific characteristics that influence consumer preferences. The first two main components (PC1 and PC2) explained 99.13% of the variance of the sensory parameters evaluated. The principal component 1 is represented by the cheese texture and the principal component 2 by the cheese flavor (Table 6).

**Figure 2:**
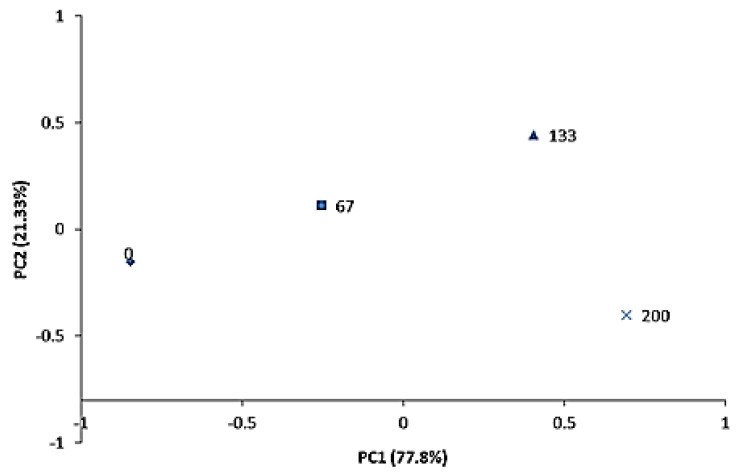
Principal components to describe the sensory profile of Minas Frescal cheese produced by lactating goats fed diets with increasing levels of licury cake. PC1, principal component 1; PC2, principal component 2.

The distribution of the treatments within the first principal component indicates that the treatments with LC inclusion of 133 and 200 g/kg DM had a more pleasant texture compared to the treatments with less inclusion. As for main component 2, cheese flavor was preferred when LC inclusion was 67 and 133 g/kg DM (Figure 2).

## 4. Discussion

### 4.1. Nutritional Components Intake and Milk Chemical Composition

The dry matter intake directly affects the intake of the other nutritional components of the diet, which may explain the similarity in CP intake. Although LC is a fibrous feed (64% NDF), the physical form in which it was added to the diet (ground) did not affect feed degradability or ruminal passage rate, so it did not affect DM intake. Similar behavior on DM and CP intakes was observed by Bagaldo et al. [7], when evaluating increasing levels of LC in diets for feedlot lambs. It is important to note that the palatability of LC was not a limiting factor for dietary intake in lactating goats.

Contrary to our results, in Silva et al. [11] and Silva et al. [6], dry matter and crude protein intake decreased in cull cows and goat kids, respectively. However, those results probably correlate with the high-concentrate diet (20:80—forage:concentrate) these animals received compared to the 50:50 diet used in the current experiment. Therefore, it is possible to infer that there is a limitation in the use of LC in high-concentrate diet for ruminants.

The chemical composition of milk is directly proportional to nutrient intake [34]. Therefore, the similarity in DM intake resulted in similar nutrient availability in the ruminal and post-ruminal environments. Consequently, the chemical composition of milk was similar between treatments, regardless of the LC inclusion in the diet. Ferreira et al. [3] observed that the inclusion of LC of up to 144 g/kg DM in the supplement for grazing lactating cows did not affect the milk chemical composition. On the other hand, it is possible to hypothesize that the proximal composition of the product is not usually affected by the nutritional conditions of diets without an excess of a specific nutrient, as observed in Silva et al. [13] and Silva et al. [6]. In these studies, the proximal composition of meat has not been altered by the inclusion of LC in the diet of beef cattle and goat kids, respectively.

### 4.2. Fatty Acid Pofile of Milk

Short- and medium-chain fatty acids are synthesized in the mammary gland from acetate and β-hydroxybutyrate compounds produced in the rumen [35]. As the similarity in nutrient intake is reflected in similar ruminal fermentation, it was also expected that fatty acid content would be similar in the current study.

The linear increase in lauric (12:0) and myristic (14:0) acid content was due to the higher proportion of these fatty acids in LC, which contains, on average, 44% lauric (12:0) and 13% myristic (14:0) acid [36].

The increase in lauric and myristic acids in meat (*Longissimus lumborum* muscle) was also observed by Costa et al. [14]. The authors evaluated the inclusion of up to 240 g/kg DM in the diet of feedlot lambs. The linear increase in lauric acid in milk was also reported by Porto Junior et al. [37] when LC was included up to 165 g/kg DM in the diet.

The importance of lauric acid in milk is positive, considering that the intake of medium chain fatty acids (MCFA) has been reported to promote benefits for human health. CMFA are more rapidly digested and absorbed due to their small size, which is a favorable feature, as these are also used as an energy source [38].

The increase in the concentration of rumenic acid (18:2 cis9trans11) in milk with the inclusion of 10.1 g of LC/kg of DM in the diet is related to the greater activity of the enzyme delta-9-desaturase, since it is known that 64% of the conjugated linoleic acid (CLA) present in milk comes from the desaturation of vaccenic acid (18:1 trans11) in the mammary gland [39].

Among the isomers of conjugated linoleic acid, rumenic acid (18:2 cis9trans11) predominates in the milk of ruminant animals. This fatty acid is correlated with the prevention of the appearance of cancer and coronary diseases [40]. Therefore, the increase in rumenic acid with the inclusion of 10.1 g of LC/kg DM in the diet indicates an improvement in the quality of milk fat.

The fatty acids that were significant in the goat milk of the current experiment were also significant in the meat fatty acid profile of goat kids in the study by Silva et al. [6]. However, the fatty acid profile was not affected in meat from beef cattle [13]. Therefore, it is important to note that rumen lipid metabolism is more marked in small ruminants. In this sense, it is important to take into account the level of LC inclusion that will improve the fatty acid profile considering human health.

### 4.3. Quality of Minas Frescal Cheese

The quadratic effect observed for cheese moisture was concave, while the curve for cheese EE content was convex, indicating an inverse relationship between these variables. The opposite behavior of these two variables can be attributed to the higher enzymatic activity of lipoprotein lipases and esterases (enzymes used for cheese production) in the moister cheeses, which probably favored lipolysis, which was reflected in the EE content [41,42].

Some factors can interfere with the yield of the cheese, among which we can mention the chemical composition of the milk, the methodology used, the activity of the enzymes, among others [43]. Consequently, the similarity in cheese yield was due to the similarity in the chemical composition of the milk between the diets studied as well as the control of the production process.

### 4.4. Sensorial Analysis

The use of multivariate methods such as principal component analysis (PCA) had the purpose of being able to observe the results from another perspective. In the current study, this analysis indicated that the first two principal components explained 99.13% of the total variation of the sensory analysis of Minas Frescal cheese. The attributes with the greatest influence on the sensory evaluation of the cheese were texture (Principal Component 1) and flavor (Principal Component 2).

It was observed that the inclusion of LC at the levels of 133 and 200 g/kg DM improved the texture of the cheese. On the other hand, the intermediate levels of LC inclusion, 67 and 133 g/kg DM resulted in a more pleasant cheese flavor, and, consequently, a higher global acceptance.

Childs and Drake [44] reported that reduction in cheese fat content is associated with changes in texture, with reductions in cheese chewiness, hardness, and elasticity, reflecting product acceptability to consumers. Therefore, the observed increase in the fat content of the cheeses due to the LC inclusion was reflected in a higher score for the cheese texture parameter.

It is known that changes in the cheese chemical composition are reflected in the flavor. These differences are mainly related to the occurrence of reactions such as proteolysis, lipolysis and carbohydrate fermentation. However, the presence of phenolic compounds is also associated with sensory changes. Phenolic compounds in adequate concentrations in milk and its derivatives have a positive relationship with the sensory quality of the product; however, in excessive concentrations it negatively affects cheese flavor and odor [45]. The licury cake has phenolic compounds, such as epicatechia, catechin, procyanidin B1 and B2, and luteolin, among others, which can influence the sensory acceptance of cheese when goats are fed with this by-product. However, the most probable hypothesis is that the intermediate levels of inclusion of LC (67 and 133 g/kg) increased acceptance of cheese flavor due to the presence of these phenolic compounds and how they are sensed by the taste buds. On the other hand, the higher inclusion level of LC (200 g/kg DM) promoted a higher concentration of phenolic compounds, which negatively affected the cheese flavor.

On the other hand, it is observed that unsaturated fatty acids, myristoleic and rumenic acids, increased linearly in the milk that was used to make cheese following LC inclusion. The increase in these fatty acids is likely to improve the flavor characteristics of the cheese; however, in excess, these fatty acids promote a certain degree of oxidation that promotes a rancid taste unpleasant for the consumer.

### 4.5. Lipids and Human Health

In recent decades, humans have been very concerned about nutrition, mainly the lipid content of the diet. This concern is due to the content of saturated and *trans* FA, which are highly correlated with the development of cardiovascular, metabolic, physiological, and even psychological diseases, directly or indirectly [46]. According to Sacks et al. [47], replacing the SFA content with PUFA can decrease the incidence of cardiovascular disease (CVD) by 30%.

Ruminant products are sources of SFA and *trans* FA, and for these reasons ruminant products are being censored in human nutrition [48]. As observed in the present study, the SFA (68.9%) and MUFA (26.6%) FAs presented the highest proportion of the total FAs. It is important to point out that LC inclusion in the diets does not influence this content. However, LC inclusion increases the fat content of cheese produced from the milk of lactating goats fed diets containing increasing levels of LC. Therefore, although the fatty acid profile is similar in the unprocessed material (milk), the different content of fat in the final product (cheese) can consequently affect the fatty acid content. This consideration is important because some studies conclude that the content of SFA and *trans* FA and its correlation with diseases is not related to a specific content of nutrients, rather it is important to consider the general distribution of macronutrients [49].

## 5. Conclusions

As licury cake inclusion did not affect cheese yield or feed intake, based on the parameters studied, the inclusion of LC of up to 133 g/kg DM in the diet is recommended, since it preserves the maximum sensorial quality of the cheese produced from the milk of feedlot lactating goats.

## Figures and Tables

**Table 1:** Proportion of ingredients and analyzed chemical composition of the diets including licury cake.

Variable	Licury Cake (g/kg)				Licury Cake
	0	67	133	200	
**Ingredient (g/kg)**					
Maize silage	500.0	500.0	500.0	500.0	-
Licury cake	0.0	67.0	133.0	200.0	-
Ground corn	225.0	183.0	146.0	108.0	-
Cottonseed meal	200.0	175.0	146.0	117.0	-
Corn germ	17.0	17.0	17.0	17.0	-
Soybean meal ^1^	42.0	42.0	42.0	42.0	-
Urea	8.0	8.0	8.0	8.0	
Mineral supplement ^2^	8.0	8.0	8.0	8.0	-
**Chemical composition (g/kg DM)**					
Dry matter (g/kg as-fed)	620.0	620.7	621.3	621.8	913.2
Mineral matter	34.0	33.0	30.5	35.1	32.1
Crude protein	184.6	184.2	182.8	178.0	176.6
Neutral detergent Fiber ap ^3^	382.6	411.8	440.0	468.3	637.2
Acid detergent Fiber ap ^3^	258.8	279.4	299.1	318.8	487.4
pdNDF ^4^	223.2	240.1	256.4	272.7	368.8
Neutral detergent insoluble nitrogen	-	-	-	-	13.7
Acid detergent insoluble nitrogen	-	-	-	-	3.1
Lignin	81.5	90.2	98.7	107.1	218.2
Ether extract	40.6	39.4	38.0	36.6	33.9
Non-fibrous carbohydrates	374.5	323.3	299.6	273.0	120.2
Total digestible nutrients	768.7	735.6	725.9	710.7	610.6

^1^ The soybean meal protein content was 48.33% DM basis; ^2^ provides per kilogram of active element: calcium—183.00 g, phosphorus—60.00 g, potassium—28.00 g, sulfur—16.00 g, magnesium—20.00 g, copper—250.00 mg, cobalt—30.00 mg, chromium—10.00 mg, iron—250.00 mg, iodine—70.00 mg, manganese—1500.00 mg, selenium—30.00 mg, zinc—3,500,00.00 mg, fluorine (max.)—600.00 mg. ^3^ Corrected for ash and protein; ^4^ potentially digestible neutral detergent fiber.

**Table 2:** Nutritional components’ intake and chemical composition of milk from lactating goats fed diets with increasing levels of licury cake.

Variable	Licury Cake (g/kg)				Mean	SEM ^1^	*p*-Value	
	0	67	133	200			Linear	Quadratic
	Nutritional component intake (kg/day)							
Dry matter	1.60	1.65	1.59	1.61	1.61	0.05	0.876	0.591
Crude protein	0.30	0.31	0.29	0.29	0.30	0.01	0.392	0.714
	Milk chemical composition (g/day)							
Fat	38.01	40.50	40.65	38.40	39.39	2.27	0.670	0.185
Protein	34.62	33.52	34.73	32.79	33.92	2.16	0.617	0.710
Lactose	46.27	44.63	47.16	43.92	45.50	3.18	0.738	0.614
Total solids	126.82	126.61	130.36	122.29	126.52	8.04	0.854	0.377
Casein	2.61	2.52	2.54	2.51	2.55	0.29	0.129	0.437

^1^ SEM, Standard error of the mean.

**Table 3:** Fatty acids of milk from lactating goats fed diets with increasing levels of licury cake.

Variable	Licury Cake (g/kg)				SEM ^1^	*p*-Value	
	0	67	133	200		Linear	Quadratic
	Fatty acid (mg/100 mg)						
Butyric acid (C4:0)	1.22	1.36	1.33	1.40	0.11	0.540	0.850
Caproic acid (C6:0)	1.86	1.83	1.82	1.71	0.10	0.567	0.823
Caprylic acid (C8:0)	2.00	2.12	2.01	1.90	0.12	0.747	0.676
Capric acid (C10:0)	6.55	7.26	6.38	6.17	0.42	0.627	0.620
Lauric acid (C12:0) ^2^	2.78	3.70	4.00	4.47	0.21	0.002	0.459
Myristic acid (C14:0) ^3^	7.53	9.30	8.77	9.53	0.57	0.001	<0.001
Myristoleic acid (C14:1) ^4^	0.07	0.09	0.10	0.16	0.02	0.002	0.177
Pentadecanoic acid (C15:0)	0.77	0.85	0.80	0.83	0.06	0.673	0.731
Palmitic acid (C16:0)	25.60	26.97	25.69	27.02	0.44	0.481	0.987
Palmitoleic acid (C16:1)	0.64	0.73	0.89	1.10	0.11	0.134	0.776
Heptadecanoic acid (C17:0)	0.78	0.51	0.14	0.17	0.09	0.005	0.326
Stearic acid (C18:0)	15.17	12.03	14.25	12.03	0.54	0.105	0.624
Vaccenic acid (C18:1 t11)	1.18	0.98	1.07	1.18	0.06	0.870	0.242
Oleic acid (C18:1 n9)	23.36	21.55	21.58	21.95	0.63	0.407	0.431
Linoleic acid (C18:2 n6)	3.86	3.65	3.46	2.93	0.20	0.130	0.708
Arachidic acid (C20:0)	0.27	0.25	0.27	0.26	0.01	0.888	0.761
Linolenic acid (C18:3 n3)	0.22	0.08	0.13	0.17	0.10	0.662	0.176
Rumenic acid (C18:2 c9t11) ^5^	0.36	0.32	0.30	0.37	0.03	0.913	0.018
Arachidonic acid (C20:4 n6)	0.19	0.28	0.18	0.23	0.26	0.936	0.756
Eicosapentaenoic acid (C20:5 n3)	0.03	0.04	0.05	0.02	0.01	0.653	0.371

^1^ SEM, Standard error of the mean; Regression equations: ^2^ C12:0 = 2.93 + 0.0081LC, R^2^ = 0.95; ^3^ C14:0 = 7.66 + 0.0304LC – 0.00012LC^2^, R^2^ = 0.42, ^4^ C14:1 = 0.06569 + 0.000407LC, R^2^ = 0.83; ^5^ C18:2 c9t11 = 0.3660–0.00135LC + 0.0000668LC^2^, R^2^ = 0.88.

**Table 4:** Fatty acid profile of milk from lactating goats fed diets with increasing levels of licury cake.

Variable	Licury Cake (g/kg)				Mean	SEM ^1^	*p*-Value	
	0	67	133	200			Linear	Quadratic
	Fatty acid profile (mg/100 mg)							
Polyunsaturated fatty acids (PUFA)	4.45	4.67	4.07	3.57	4.19	0.26	0.189	0.502
Monounsaturated fatty acids	26.26	24.25	24.51	25.23	25.06	0.70	0.678	0.376
Saturated fatty acids (SFA)	64.17	66.11	65.89	63.03	64.80	0.83	0.517	0.631
PUFA:SFA	0.07	0.07	0.06	0.05	0.06	0.00	0.189	0.581
Total	94.88	95.04	94.43	94.84	94.80	0.20	0.701	0.769
Omega-6 (ω-6)	3.86	3.65	3.46	2.93	3.48	0.20	0.130	0.708
Omega-3 (ω-3)	0.23	0.08	0.25	0.17	0.18	0.10	0.983	0.747
ω-6:ω-3	25.88	22.84	29.16	18.57	24.11	4.73	0.741	0.721
Conjugated linoleic acid	0.36	0.37	0.36	0.37	0.37	0.03	0.815	0.073
Atherogenicity index	2.16	2.53	2.59	2.37	2.41	0.12	0.562	0.304
Thrombogenicity index	3.09	3.11	3.34	3.16	3.18	0.13	0.321	0.628
hypocholesterolemic:hypercholesterolemic ratio	0.75	0.76	0.64	0.63	0.70	0.02	0.449	0.420

^1^ SEM, Standard error of the mean.

**Table 5:** Chemical composition and yield of cheese made from the milk of lactating goats fed diets with increasing levels of licury cake.

Variable	Licury Cake (g/kg)				SEM ^1^	*p*-Value	
	0	67	133	200		Linear	Quadratic
	Chemical composition (% DM)						
Moisture ^2^	60.66	56.32	53.31	57.74	1.13	0.009	0.001
Crude protein	44.55	45.14	43.56	43.58	0.42	0.104	0.263
Ether extract ^3^	40.33	44.53	46.69	45.66	0.74	0.002	0.021
Ash ^4^	7.64	6.32	6.28	5.57	0.21	<0.001	0.300
	Kg of cheese/100 kg of Milk						
Yield	16.66	16.44	15.18	16.13	0.19	0.052	0.068

^1^ SEM, Standard error of the mean; Regression equations: ^2^ Moisture = 61.0255 − 0.1221LC + 0,000523LC^2^, R^2^ = 0.69, ^3^ Ether extract = 41.3017 + 0.074266LC − 0.00024LC^2^, R^2^ =0.21, ^4^ Ash = 7.40–0.01LC, R^2^ = 0.88.

**Table 6:** Main components related to the sensory evaluation of Minas Frescal cheeses produced from lactating goats fed diets with increasing levels of licury cake.

Principal Component	Eigenvalues	Variance Ratio (%)	Cumulative Ratio (%)	Color	Odor	Flavor	Texture	Global Acceptance
CP1	0.476	77.800	77.800	0.151	0.192	−0.292	0.9221	0.069
CP2	0.130	21.330	99.130	−0.003	0.237	0.764	0.1497	0.580
CP3	0.005	0.870	100.000	−0.377	0.851	0.020	−0.0827	−0.354
CP4	0.000	0.000	100.000	0.914	0.321	0.059	−0.1863	−0.156
CP5	0.000	0.000	100.000	0.000	0.282	−0.573	−0.293	0.713

## Data Availability

Not applicable.
